# Weight Gain in Family-Based Treatment for Avoidant/Restrictive Food Intake Disorder (ARFID) with and Without Autism

**DOI:** 10.1007/s10578-025-01810-w

**Published:** 2025-01-30

**Authors:** Cathrine Terese Lien, Nicola Reichel, Nadia Micali, Mette Bentz

**Affiliations:** 1 Child and Adolescent Mental Health Center, Copenhagen University Hospital– Mental Health Services CPH, Copenhagen, Denmark; 2 Center for Eating and Feeding Disorders Research (CEDaR), Mental Health Center Ballerup, Copenhagen University Hospital - Mental Health Services CHP, Copenhagen, Denmark; 3 Great Ormond Street Institute of Child Health, University College London, London, UK

**Keywords:** ARFID, Autism, Children, Adolescents, Family-based treatment, Underweight

## Abstract

Health-threatening underweight and poor growth is a frequent consequence of Avoidant restrictive food intake disorder (ARFID) and should be a priority for treatment in children and adolescents (young people, YP). Coexisting autism is more prevalent in YP with ARFID than in YP with other eating disorders. Treatment studies are still sparse for ARFID, and it is not known whether treatment response is lower in those with coexisting autism, as it is documented in other eating disorders. In this study we examined if family-based treatment for ARFID (FBT-ARFID) was associated with weight gain in underweight young people (YP) with ARFID and if coexisting autism affected weight gain. A clinical naturalistic prospective case series of 33 YP aged 6.3–18 years with ARFID presentations and underweight were offered a manualized FBT-ARFID with weight monitoring. We examined changes in body weight between start and end of treatment in those with and without diagnosed coexisting autism. The majority of participants (*N* = 26, 79%) had a weight gain between 1 and 15 kg, whereas 7 YP (21%) had a stable body weight between start and end of treatment (differences between − 0.6 kg and + 0.9 kg). At the group level, weight gain was statistically significant between start and end (Median = 3.9 kg (4.0, -0.6 -15.1) z = 4.491, *p* <.001). There was no significant difference in weight gain between participants with (*N* = 14, 42%) and without (*N* = 19, 58%) coexisting autism. Participants had a significant weight gain at the group level, suggesting that FBT-ARFID is associated with weight gain in the majority of underweight ARFID patients, both with and without coexisting autism. However, a subgroup may need additional interventions in order to secure weight rehabilitation.

## Introduction

Avoidant/restrictive food intake disorder (ARFID) is a relatively new diagnosis introduced in the fifth edition of Diagnostic and statistical manual of mental disorder (DSM-5) in 2013 and in the International Classification of Diseases 11th version (ICD-11) in 2022 [1, 2]. ARFID is a serious eating disorder characterized by avoidant and/or restrictive eating that are not driven by concerns about weight and body image as is the case for anorexia nervosa (AN) and bulimia nervosa (BN). Individuals with ARFID avoid certain types of food or limit their intake of food due to a range of other reasons e.g., sensory sensitivity, concern about aversive consequences and/or lack of interest in food [3]. To fulfill criteria for ARFID the failure to meet nutritional needs must have serious consequences, such as: malnutrition, failure to thrive, weight loss, psychosocial impairment and/or dependence on supplemental feeding [1].

Due to the shorter history of the ARFID diagnosis treatment studies are still limited. For AN children and adolescents (young people, YP), Family-based Treatment (FBT) is recommended first line of treatment [4, 5]. For ARFID in YP, family-based Treatment for ARFID (FBT-ARFID) and cognitive behavior therapy for ARFID (CBT-AR) [3] are promising treatments The two approaches have many similarities but differ regarding who has a leading role for change (parents vs. young person (YP)) [3]. FBT-ARFID has been tested in a feasibility trial with 14 participants in the FBT-ARFID arm, and the study found higher weight gain in FBT-ARFID than usual care [6].

We will use the term autism for the broad spectrum of autism spectrum conditions, in line with recommendations from Autism Europe [7]. Individuals with autism are at higher risk of developing ARFID. Inoue T. et al. found the prevalence of autism in an ARFID population to be as high as 12,5% [8] The research on individuals with anorexia nervosa indicates that co-existence of autism influences the course of treatment: a high level of autism features was associated with more severe presentations in one study [9] and with more frequent need of hospitalization in other studies [10, 11]. Moreover, in a population-based follow-up study of YPs with anorexia nervosa in adolescence, coexisting autism was associated with more dietary restraint and reduced mental health at 30-year follow-up, compared with those with anorexia nervosa in adolescence and no autism [12]. Considering the high prevalence of autism in ARFID populations it is important to understand the role of autism in ARFID treatment and to assess efficacy of treatment when coexisting autism is present. The differential outcome of YPs with coexisting autism to FBT-ARFID is not reported. The weight loss and/or faltered growth that often is associated with ARFID has serious consequences for the development and well-being of YP, and in these cases, any treatment approach should focus on securing a stable weight gain and reestablish growth.

Consequently, we aimed to examine if FBT-ARFID is associated with weight gain in underweight YP with ARFID, and if weight gain is different in YP with coexisting autism compared with those without autism.

## Method

Potential participants were children and adolescents between 6 and 18 years old, that were referred with an ARFID-presentation to a specialized outpatient clinic for YP with eating disorders between May 2018 and November 2023 in the Capital Region of Denmark (*N* = 60). FBT-ARFID was offered if diagnostic criteria for ARFID were met and a somatic cause for the disturbed eating was ruled out or treated (*N* = 53). YP in need of gastric tube feeding or intensive daily treatment are not routinely offered outpatient treatment in this setting and were therefore not included in the study.

Of those starting FBT-ARFID, 43 (81%) gave informed consent to a prospective clinical study monitoring response to treatment.

Since our present focus was weight gain between start and end of FBT-ARFID, we excluded those participants who were still in treatment by the time of the study (*N* = 3), and those who were not in need of weight gain (*N* = 7) but had other serious health consequences of their eating pattern, resulting in a study sample of 33 participants. Descriptive information is presented in Table 1.

**Table 1:** Descriptive information of study sample and subgroups with and without autism

	Study sample, *N* = 33	Study sample without autism, *N* = 19	Study sample with autism, *N* = 14	Group difference no autism/autism
Age at start of treatment, mean (SD, range)	12.5 (2.9, 6.8–17.8)	13.1 (2.7, 7.8–17.8)	11.6 (2.9, 6.8–15.8)	Independent samples t(31)= -1.447, *p* =.16
Female sex, N (%)	16 (48.5)	8 (42.1)	8 (57.1)	Pearson Chi-Square test of proportions *p* =.49
Body weight in kg, start of treatment, mean (SD, range)	32.4 (9.9, 13-52.8)	33.4 (9.5, 17.1–52.8)	31 (10.6, 13-45.2)	Independent samples t(31) = − 0.699, *p* =.49
Body weight in kg, end of treatment, mean (SD, range)	36.3 (11.9, 15-67.9)	37.6 (11.6, 18.4–67.9)	34.5 (12.4, 15-53.2)	Mann-Whitney U-test: mean rank 17.7/ mean rank 16; U = 146.5, z = 0.492, *p* =.63
Weight gain during treatment in kg, mean (SD, range)	3.9 (4.0, (-0.6) -15.1)	4.2 (4.0, 0.4–15.1)	3.5 (3.9, (-0.6) -13.5)	Mann-Whitney U-test: mean rank 17.9 / mean rank 15.8; U = 150.5, z = 0.638, *p* =.53
Height in cm, start of treatment, mean (SD, range)	149.5 (20.1, 108–187)	150.8 (18.6, 110.4–187)	147.7 (22.5, 108–176)	Mann-Whitney U-test: median 154/ median 150.5; U = 140, z = 0.255, *p* =.82
Height in cm, end of treatment, mean (SD, range)	151.1 (19.2, 109–187)	152.9 (17.5, 118–187)	148.8 (21.8, 109–176)	Independent samples t(31) = − 0.593, *p* =.56
Height growth during treatment in cm, mean (SD, range)	1.67 (1.9, 0-7.6)	2.1 (2.2, 0-7.6)	1.1 (1.4, 0-4.4)	Mann-Whitney U-test: mean rank 18.6/ mean rank 14.9; U = 162.5, z = 1.11, *p* =.29
% of median BMI for age and sex ǂ, start of treatment, mean (SD, range)	77.7 (6.8, 66.1–94.7)	78.1 (7.7, 66.1–94.7)	77.2 (5.5, 69.4–86.9)	Independent samples t(31) = − 0.383, *p* =.71
% of median BMI for age and sex ǂ, end of treatment, mean (SD, range)	84.1 (7.8, 70.6-102.3)	84.7 (7.7, 70.6-102.3)	83.4 (8.0,72.3–99)	Independent samples t(31) = − 0.475, *p* =.64
Duration of treatment in months, mean (SD, range)	5.1 (4.0, 0.5–21)	4.3 (2.9, 0.5–11)	6.1 (5, 1–21)	Mann-Whitney U-test: mean rank 15.7/ mean rank 18.7; U = 109, z = − 0.882, *p* =.40
Number of treatment sessions #	8.2 (3.6, 3–15)	8 (3.7, 3–13)	8.7 (3.7, 4–15)	Mann-Whitney U-test: mean rank 12.4/ mean rank 14; U = 63, z = − 0.517, *p* =.64

SD = standard deviation

ǂ: According to Danish population norms [8]

#: Missing information on number of sessions for 3 participants with autism and 5 participants without

### Assessment

The ARFID diagnosis was evaluated by a multi-disciplinary team in diagnostic interviews with YP and parents (The Pica, ARFID & Rumination Disorder Interview [13]) and a somatic evaluation by a medical doctor. Information on patients’ earlier growth history was collected.

The presence of coexisting autism was based on clinical diagnosis established prior to commencement of FBT-ARFID.

The assessment of need for weight gain was based on a clinical evaluation by a physician, based on the earlier growth trajectory of the YP, or in cases of stagnated height growth (where weight for height may appear close to normal but the haltered height growth indicate undernutrition), the assessment was based on securing sufficient weight to catch up on expected height growth and ensure puberty development. As these YP had often been eating small amounts for many years of life many had followed a growth curve at the bottom of or below the normal distribution the goal was not to normalize weight for height but to set the YP on a stable trajectory to increase weight and equip the families with strategies to maintain weight gain, Consequently, total weight gain in kg during treatment was our chosen outcome, but % of median BMI for age and sex is reported in Table 1 for additional information.

### Treatment

Families were offered a course of FBT-ARFID planned for up to 13 sessions. Number of sessions was not fixed but decided in collaboration between treatment team and family, and it might end before 13 sessions if e.g., parents were able to continue the work on their own after psychoeducation and introduction to the main principles of gradual exposure, or if they felt it was less useful or too stressful for the family. FBT-ARFID was based on a locally developed guideline informed by a cognitive-behavioral approach to gradual exposure along with principles of FBT for AN, i.e., delivered in a family therapy setting, focusing on psychoeducation, empowering of parents; externalization of the disorder; and an emphasis of behavioral change. The clinical guideline was developed prior to the publication of the manual by J. Lock [14, 15].

The primary focus of FBT-ARFID for patients with underweight was weight gain to ameliorate nutritional deficiencies. Secondary goals were variation, flexibility, and age-appropriate independence in eating.

For FBT-ARFID, psychoeducation for parents is especially important as the eating difficulties are expected to be long-standing, and parents need strategies to keep developing and maintaining the child´s eating behaviors until the child is able and mature enough to take responsibility for his/her own health. Psychoeducation consisted of introduction to ARFID and the different ARFID-profiles, consequences of malnutrition, maintaining factors and normal eating development in children. Homework between sessions is agreed on, and parents are encouraged to secure continuous exposure as well as reward and emotional support.

The FBT-ARFID sessions begin with getting an overview of food eaten, using categories (always eats, sometimes eats, and never eats). The family is guided to increase the amount of food on the “always-list” to establish weight gain. Next step is to introduce the families to gradual exposure work corresponding to the individual ARFID-profile [14, 15]. The ARFID profiles are not mutually exclusive, and the same patient can have symptoms from more than one ARFID profile (sensory sensitivity, concern about consequences, and/or lack of interest). The food targeted for exposure is chosen collaboratively, with nutrition and exposure difficulty in mind. A meal is held in-session, where coping strategies for exposing situations can be practiced and discussed.

Progress is monitored in each session, both by collecting reports from the family, making updates of the list (always, sometimes, never) and measure weight/height.

In children with high rigidity and difficulties in emotional tolerance, as is often the case in autism, goals sometimes need to be adjusted and exposure must be planned in smaller steps to increase YP participation, success, and motivation. If the child was not able to participate in sessions and exposure planning due to e.g., anxiety, parents were offered parent-sessions with a focus on psychoeducation, how to work with exposure and emotional support, giving them the basic techniques, that they could then later practice in everyday life at home and thereby help their child.

### Statistical Analyses

Data were analyzed using SPSS v.29. There were three outliers with large weight gain values, and the distribution of weight change between start and end of treatment was not normally distributed. Therefore, a Wilcoxon Signed Rank Test was run to analyze the difference between time points. An independent Mann-Whitney U-test was used to compare weight change in participants with and without a diagnosed autism spectrum condition. One of the advantages of these tests is that they are not sensitive to outliers as the ranks data points before analysis.

## Results

A total of 14 participants (42%) had coexisting autism. FBT-ARFID lasted between 3 and 15 sessions with a mean duration of 4.7 months.

**Fig. 1:**
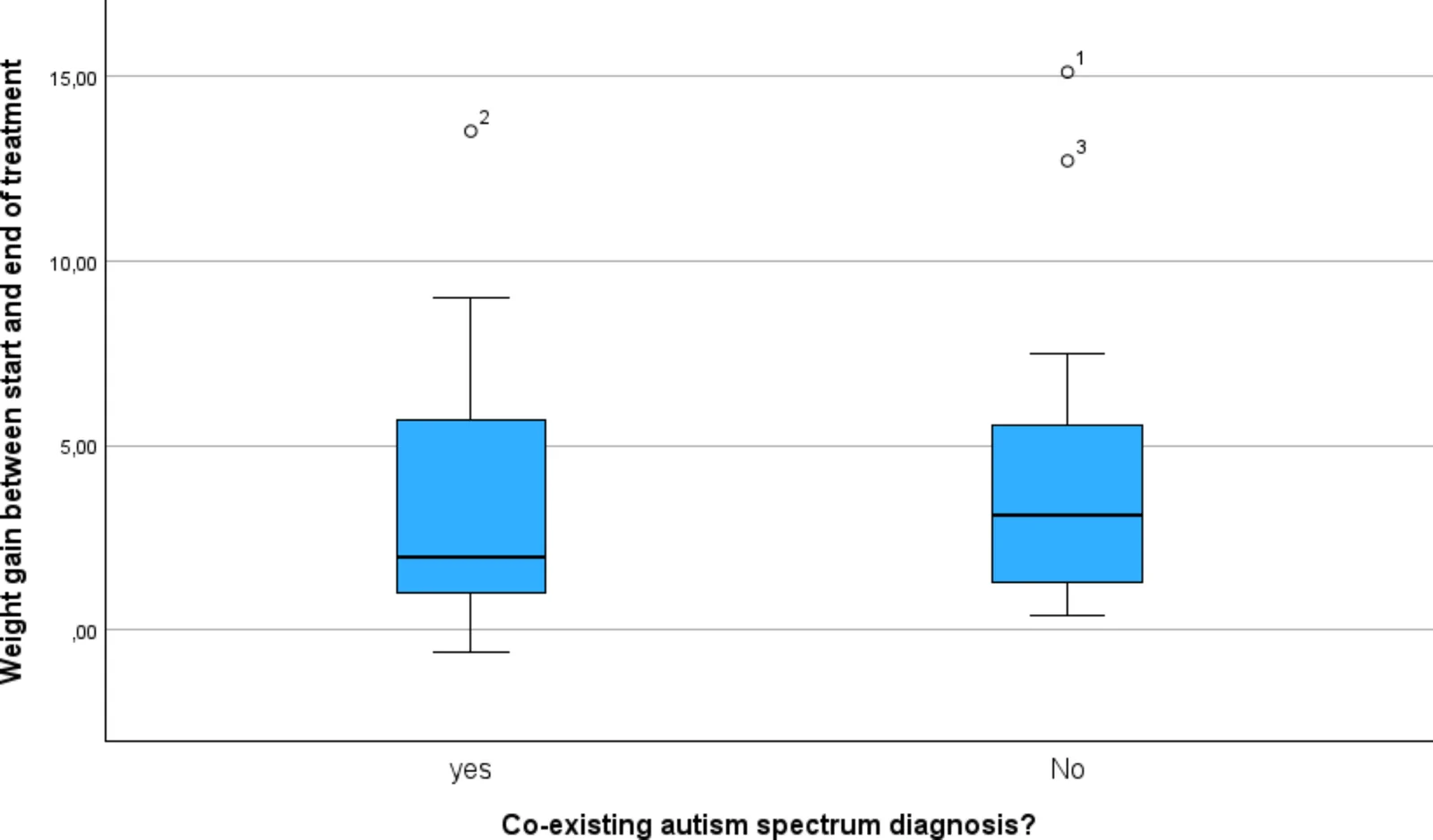
Box plot of weight gain between start and end of treatment for participants with and without co-existing autism spectrum condition

Three participants (9%) gained between 10 and 15 kg between start and end of treatment, 23 participants (70%) had weight gains between 1 and 9 kg, whereas seven (21%) had no difference in body weight (differences between − 0.6 kg and + 0.9 kg). Median weight at the start of treatment was 31.75 kg; median weight by the end of treatment was 35.9 kg; median weight gain was 2.9 kg (interquartile range = 4.8 kg) (Fig. 1). At the group level, median weight gain was statistically significant, z = 4.491, *p* <.001. There was no significant difference in weight gain between the underweight participants with coexisting autism.

## Discussion

This naturalistic study aimed to investigate if FBT-ARFID for underweight YP with ARFID would lead to a significant weight gain, and furthermore if coexisting autism had an impact on the efficacy of the FBT-ARFID.

We found a significant weight gain at the group level, suggesting that an FBT-ARFID treatment course of up to 13 sessions is associated with weight gain in YP with stagnated growth and/or weigh loss prior to treatment. We found no significant differences between patients with or without autism. Our findings are consistent with earlier studies which have documented weight gain following FBT-ARFID [6, 16, 17], and our findings demonstrate that this is true for YP with coexisting autism as well. In extension, our findings support the treatment model where parents are the primary agents of change, i.e., where parents are empowered to intervene, modify, and support the eating pattern of their YP is efficacious. The manual in use suggests that the pace and steps in the gradual exposure work is adapted to the individual YP, focus is on repetition of steps to secure that progress may be sustained over time, rather than securing rapid weight recovery as would be a goal of FBT for AN. We speculate that the parent-led approach may help secure an individually tailored pace which may have been especially important for the YPs with coexisting autism, as their parents often have years of experience with how to implement change, guide their child through transitions, and cope with frustration. We suggest that there is a great potential in FBT-informed approaches when this unique knowledge of parents is activated and coupled with information on eating and gradual exposure and used to counter an eating disorder threatening their child´s health. Other treatment approaches involving parents only, such as the SPACE-ARFID are other examples of recruiting this parental resource as the primary agent of change [18, 19].

The large differences in weight gain within the sample indicate that not all participants were set on trajectory to increase their energy intake and restore their body weight despite FBT-ARFID and parental involvement. This finding points to the need to better understand characteristics of this group, and to identify other forms and/or intensity of treatment to meet the needs of this subgroup.

In addition, there is a need for studies on the treatment of ARFID when comorbidities other than autism are present. Kambanis et al. documented comorbidity in nearly half of individuals with ARFID, and they suggested similar sensory processing difficulties in individuals with ADHD as in individuals with autism [20]. These findings illustrate the heterogeneity among individuals with ARFID presentations and point towards a need for differential treatment approaches.

### Strengths and Limitations

This study has several strengths, including the manualized nature of the treatment, therapists were trained and received supervision, and the sample represented all referred YPs within a geographical area. Relevant limitations are the small sample size, the lack of a comparison group, variability in the number of sessions, and lack of measurement of aspects of outcome and improvement other that weight gain. Moreover, there is a large variability in number of sessions, but we were not able to examine the individual reasons for terminating treatment at specific time points. Further, we are not able to report on the specific ARFID presentations of the sample (e.g., sensitivity, concern, lack of interest or combinations thereof), and therefore we are not to ascertain if weight gain was equally distributed across presentations. Lastly, we lack information on how many times a YP did not attend all sessions, and these points add an unfortunate variability, that may have impacted on outcomes, affecting comparisons with studies on the now published FBT-ARFID [15]. Additional research is needed to examine the process of improvement and the effectiveness of family-based interventions on other features of the disorder. Moreover, follow up may be especially relevant in ARFID as the profiles that lead to undereating in the first place may be life-long, and it is especially important that the YP and families are able to maintain the strategies they implemented during FBT to prevent relapse.

## Conclusion

ARFID-focused FBT is effective in securing weight gain in YP with ARFID with and without co-existing autism. Larger randomized studies and long-term follow-up studies are needed to examine the overall effect of FBT-ARFID, the differential effect for different ARFID profiles, and its effectiveness when another mental comorbidity than autism is present.

## Data Availability

The dataset analyzed during the current study is available in anonymizedform from the corresponding author on reasonable request.
